# Psychiatric Morbidities in Patients with Non-communicable Diseases among Inpatients of Medicine Department in a Tertiary Care Hospital: A Descriptive Cross-sectional Study

**DOI:** 10.31729/jnma.5255

**Published:** 2020-12-31

**Authors:** Kalpana Sharma, Govinda Dhungana, Shailendra Adhikari, Archana Bista Pandey, Muna Sharma

**Affiliations:** 1 Department of Adult Health Nursing, Chitwan Medical College, Bharatpur, Chitwan, Nepal; 2 Department of Statistics, Birendra Multiple Campus, Bharatpur, Chitwan, Nepal; 3 Department of Psychiatry, Chitwan Medical College, Bharatpur, Chitwan, Nepal; 4 Department of Women Health and Development, Institute of Medicine, Kathmandu, Nepal; 5 Department of Adult Health Nursing, Institute of Medicine, Kathmandu, Nepal

**Keywords:** *Nepal*, *non-communicable diseases*, *patients*, *psychiatric morbidities*

## Abstract

**Introduction:**

Psychiatric morbidities are common among patients with chronic non-communicable diseases. These diseases have high morbidity, mortality, and higher health costs. However, psychiatric conditions are often underdiagnosed and undertreated in our country. This study aimed to find out the psychiatric morbidities among patients with non-communicable diseases admitted in inpatients units of medicine department.

**Methods:**

A descriptive cross-sectional study was conducted in inpatients of medicine department of a tertiary care hospital among 926 patients with chronic non-communicable diseases. Ethical approval was obtained from Chitwan Medical College Institutional Review Committee (Ref. No. CMC-IRC: 2074/75: 38). Convenient sampling technique was used. Patients were interviewed using the Patients Health Questionnaire. Data analysis was performed using Statistical Package for Social Sciences version 16.

**Results:**

Among 926 non-communicable diseases patients, psychiatric morbidities observed were somatization 612 (66.1%) anxiety 319 (34.4%) and depression 379 (40.9%). Patient with multiple non-communicable diseases had higher psychiatric morbidities compared to patients with a single disease.

**Conclusions:**

Psychiatric morbidities are common among admitted patients suffering from non-communicable diseases in Nepal. Hence, regular screening services are needed in all level of health care centres to identify and treat the risk groups on time.

## INTRODUCTION

Non-communicable diseases (NCDs) are the leading cause of deaths globally.^1^ It accounts for more than 85% of premature deaths occurred in low and middle-income countries.^2^ Psychiatric morbidities such as depression and anxiety are high in NCDs patients.^3,4^ They are collectively responsible for 72% of all deaths.^5^ The co-existence of psychiatric disorders with NCDs has important implications on quality of life, general well-being, cost of treatment and general longevity of the patient.^3,6^

In Nepal, NCDs accounts for 66% of deaths in 2016.^7^ The prevalence of mental disorders is higher among physically ill patients (31.7%)^8^ compared to healthy adults (13.2%).^9^ These conditions are underdiagnosed and undertreated in our country due to social stigma. Also, there is limited literature on the prevalence of mental disorders among patients with NCDs.

This study aimed to find out psychiatric morbidities among NCDs patients admitted in inpatients units of medicine department of Chitwan Medical College.

## METHODS

A descriptive cross-sectional study was conducted in inpatients of medicine department of Chitwan Medical College Teaching Hospital, Chitwan from 15th June 2018 AD to 17th September 2019 AD. Ethical approval was obtained from the Chitwan Medical College Institutional Review Committee (Ref. No. CMC-IRC: 2074/75: 38). The study population was those patients who were clinically diagnosed to have NCDs such as type-II diabetes mellitus (DM), hypertension (HTN), coronary artery diseases (CAD), chronic kidney disease (CKD) and chronic obstructive pulmonary diseases (COPD) either alone or in combination for 1 year and above and were admitted in different inpatients units of medicine department.

Sample size calculation,

$$\begin{array}{ll} \text{n} & \text{= }{\text{Z}}^{\text{2}}\times \text{p}\times (\text{1}-\text{p)}/{\text{e}}^{\text{2}}\\ & \text{= }{\left(1.96\right)}^{\text{2}}\times 0.317\times 0.683/{(\text{0.03)}}^{\text{2}}\\ & \text{= }925 \end{array}$$

Where,

n = Sample size
Z = 1.96 at 95% Confidence Interval
p = estimated prevalence rate (31.7%) = 0.317
e = Margin of error, 3%

The sample size of 925 patients was calculated considering the confidence level of 95%. Convenient sampling technique was used for recruiting the desired number of samples in the study. Structured interview schedule for the socio-demographic and disease-related information and Patient Health Questionnaire (PHQ) for the screening of psychiatric morbidities were used for the data collection. PHQ contains 3 parts: PHQ-15 to assess somatic functions, Generalized Anxiety Disorders-7 (GAD-7) to assess anxiety and PHQ-9 to assess depression. The PHQ questionnaire is a validated questionnaire, which found to be useful in the screening of patients for psychiatric illness worldwide.^4,6,10,11^ It assesses the symptoms experienced by participants during the 2 weeks before they take the survey. Each item of GAD-7 and PHQ-9 was rated 0 to 3 scores where 0-not at all, 1-several days, 2-more than half of the days, and 3-nearly every day, with higher scores indicating patients' increased self-report of anxiety and depression severity. Scores obtained in GAD-7 was classified into mild (5-9), moderate (10-14), and severe anxiety (≥15). Likewise, scores of PHQ-9 was divided into mild (5-<10), moderate (10-<15), moderately severe (15-<20) and severe (≥20) depression.

To maintain the rights of patients, further evaluation was done for those patients who were found to be positive on PHQ by psychiatrists using Standard Beck Anxiety Scale^12^ and Hamilton Depression Scale^13^ for the confirmation and further treatment. Written informed consent was obtained from the patients ensuring their confidentiality of the information. Collected data were entered into Epi data 3.1 and exported to IBM SPSS version 20. Then data were analyzed in terms of descriptive statistics such as frequency, and percentages.

## RESULTS

A total of 926 patients participated in the study. Among them, 548 (59.2%) were female and the mean age was 61.12 (±17.79) years. More than two-thirds of the patients were residing in the urban area 624 (67.4%), married 677 (73.2%) and belonged to the joint family 682 (73.7%). Nearly half of the patients were illiterate 461 (49.8%), involved in agriculture 453 (48.9%) and reported that their family income was just sufficient for the daily expenses. However, more than two-third 627 (67.7%) of the patients reported the illness impact on their job as a result they quitted their job. Regarding the behavioural pattern, nearly half 451 (48.7%) of the patients were never smoker, and 122 (13.2%) of patients were still smoking a cigarette. Only 203 (21.9%) of patients had a habit of alcohol use. Regarding clinical variables, more than one-quarter of patients had multiple NCDs 356 (38.4%) followed by COPD 188 (20.3%), diabetes mellitus 110 (11.9%) and HTN 102 (11.0%). More than half 535 (57.8%) of the patients' duration of diagnosis was less than 5 years and 532 (64.3%) of patients had a history of hospital admission in the last year (Table 1).

**Table 1:** Socio-demographic, behavioural pattern and disease-related variables of patients (n=926).

Variables	n (%)	Variables	n (%)
Age groups in years		Occupation	
< 30	31 (3.3)	Agriculture	453 (48.9)
30 - 49	148 (16.1)	Homemade work	283 (30.6)
50 - 69	444 (47.9)	Earn Cash^ε^	141 (15.2)
70+	303 (32.7)	Others	49 (5.3)
Mean ± SD= 61.12±17.79		Illness impact on job	
Sex		Yes	627 (67.7)
Female	548 (59.2)	No	299 (32.3)
Male	378 (40.8)	Family income	
Religion		Insufficient for daily expenses	229 (24.7)
Hindu	787 (85.0)	Just sufficient for daily expenses	410 (44.3)
Others^©^	139 (15.0)	Surplus for daily expenses	287 (31.0)
Caste		Food habit	
Bramin	396 (39.8)	Vegetarian	195 (21.1)
Chhetri	152 (16.4)	Non-vegetarian	731 (78.9)
Janajati	308 (33.3)	Smoking habit	
Dalit and Madhesi	97 (10.5)	Smoker	122 (13.2)
Place of residence		Past smoker	353 (38.1)
Rural	302 (32.6)	Never smoker	451 (48.7)
Urban	624 (67.4)	Alcohol intake	
Marital status		Yes	203 (21.9)
Unmarried	27 (2.9)	No	723 (78.1)
Married	677 (73.2)	Type of NCDs	
Widow /widower	218 (23.5)	Diabetes mellitus	110 (11.9)
Divorce and others	4 (0.4)	Hypertension	102 (11.0)
Family type		Coronary artery disease	97 (10.5)
Nuclear	244 (26.3)	Chronic kidney disease	73 (7.9)
Joint	682 (73.7)	Chronic obstructive pulmonary diseases	188 (20.3)
Current living status		Multiple NCDs	356 (38.4)
Alone	98 (10.6)	Duration of disease diagnosis in the years	
With family	828 (89.4)	<5	535 (57.8)
Education status		≥ 5	391 (42.2)
Illiterate	461 (49.8)	Hospital admission in the last one year	
Literate	266 (28.7)	None	331 (35.7)
Primary (1-8)	107 (11.6)	<3	481 (51.9)
Secondary and above	92 (9.9)	≥3	114 (12.3)

© Others: Buddish, Cristian Muslim, Kirat and others

ε Eam Cash: business, service and daily wage who earn direct cash

Out of 926 patients with NCDs, symptoms of somatization, depression and anxiety were observed among 612 (66.1%), 379 (40.9%) and 319 (34.4%) respectively. The higher proportion of 679 (73.3%) of patients with multiple NCDs showed evidence of psychiatric morbidities compared to patients with single NCDs. Similarly, the prevalence of psychiatric morbidities was proportionally higher among patients with DM, HTN, and CKD than patients with other NCDs (Table 2).

**Table 2:** Psychiatric morbidities among patients with NCDs.

Types of NCDs	Somatization n (%)	Anxiety n (%)	Depression n (%)
Diabetes Mellitus (n=110)	81 (73.6)	48 (43.6)	55 (50.0)
Hypertension (n=102)	73 (71.6)	29 (28.4)	32 (31.4)
Coronary artery disease (n=97)	34 (35.1)	15 (15.5)	15 (15.5)
Chronic kidney disease (n=73)	49 (67.1)	29 (39.7)	36 (49.3)
Chronic obstructive pulmonary disease (n=188)	89 (47.3)	20 (10.6)	26 (13.8)
Multiple NCDs (n=356)	286 (80.3)	178 (50.0)	215 (60.4)
Total (n=926)	612 (66.1)	319 (34.4)	379 (40.9)

Overall, the symptoms of somatization were found among 612 (66.1%) of NCDs patients where mild, moderate and severe symptoms of somatization were 360 (38.9%), 198 (21.4%), 54 (5.8%) respectively (Table 3).

**Table 3:** Level of somatization among patients with NCDs (n=926).

Types of NCDs	Level of Somatization				
	None n (%)	Mild n (%)	Moderate n (%)	Severe n (%)	Total n (%)
Diabetes Mellitus (n=110)	29 (26.4)	34 (30.9)	39 (35.5)	8 (7.3)	81 (73.6)
Hypertension (n=102)	29 (28.4)	47 (46.1)	19 (18.6)	7 (6.9)	73 (71.6)
Coronary artery disease (n=97)	63 (64.9)	26 (26.8)	8 (8.2)	0 (0.0)	34 (35.1)
Chronic kidney disease (n=73)	24 (32.9)	30 (41.1)	19 (26.0)	0 (0.0)	49 (67.1)
Chronic obstructive pulmonary disease (n=188)	99 (52.7)	75 (39.9)	8 (4.3)	6 (3.2)	89 (47.3)
Multiple NCDs (n=356)	70 (19.7)	148 (41.6)	105 (29.5)	33 (9.3)	286 (80.3)
Total (n=926)	314 (33.9)	360 (38.9)	198 (21.4)	54 (5.8)	612 (66.1)

The symptoms of anxiety were proportionally higher among patients with multiple NCDs 178 (50.0%). Among single NCDs, the symptoms of anxiety were proportionally higher in patients with DM 48 (43.6%) and CKD 29 (39.7%) as compared to other diseases. Overall, 319 (34.4%) of patients with NCDs had anxiety symptoms where 164 (17.7%), 128 (13.8%), and 27 (2.9%) had mild, moderate and severe depression respectively (Table 4).

**Table 4:** Level of anxiety among patients with NCDs (n=926).

Types of NCDs	Level of Anxiety				
	None n (%)	Mild n (%)	Moderate n (%)	Severe n (%)	Total n (%)
Diabetes mellitus (n=110)	62 (56.4)	20 (18.2)	26 (23.6)	2 (1.8)	48 (43.6)
Hypertension (n=102)	73 (71.6)	21 (20.6)	3 (2.9)	5 (4.9)	29 (28.4)
Coronary artery disease (n=97)	82 (84.5)	3 (3.1)	12 (12.4)	0	15 (15.5)
Chronic kidney disease (n=73)	44 (60.3)	15 (20.5)	14 (19.2)	0	29 (39.7)
Chronic obstructive pulmonary disease (n=188)	168 (89.4)	14 (7.4)	6 (3.2)	0	20 (10.6)
Multiple NCDs (n=356)	178 (50.0)	91 (25.6)	67 (18.8)	20 (5.6)	178 (50.0)
Total (n=926)	607 (65.6)	164 (17.7)	128 (13.8)	27 (2.9)	319 (34.4)

The evidence of depressive symptoms was higher in patients with multiple NCDs that is, 215 (60.4%) followed by diabetes 55 (50.0%) and CKD 36 (49.3%). Overall, 379 (40.9%) of NCDs patients had depressive symptoms where mild, moderate and severe depressions among patients with NCDs were 196 (21.2%), 167 (18.1%), and 16 (1.7%) respectively (Table 5).

**Table 5:** Level of depression among patients with NCDs (n=926).

Types of NCDs	Level of Depression				
	None n (%)	Mild n (%)	Moderate n (%)	Severe n (%)	Total n (%)
Diabetes Mellitus (n=110)	55 (50.0)	23 (20.9)	30 (27.3)	2 (1.8)	55 (50.0)
Hypertension (n=102)	70 (68.6)	20 (19.6)	7 (6.9)	5 (4.9)	32 (31.4)
Coronary artery disease (n=97)	82 (84.5)	12 (12.4)	3 (3.1)	0 (0.0)	15 (15.5)
Chronic kidney disease (n=73)	37 (50.7)	21 (28.8)	15 (20.5)	0 (0.0)	36 (49.3)
Chronic obstructive pulmonary disease (n=188)	162 (86.2)	13 (6.9)	13 (6.9)	0 (0.0)	26 (13.8)
Multiple NCDs (n=356)	141 (39.6)	107 (30.1)	99 (27.8)	9(2.5)	215 (60.4)
Total (n=926)	547 (59.1)	196 (21.2)	167 (18.0)	16 (1.7)	379 (40.9)

## DISCUSSION

Our study revealed that the psychiatric morbidities are common among the patients suffering from chronic NCDs such as DM, HTN, CAD, COPD and CKD. Two in three NCDs patients suffer from the symptoms of somatization whereas one in three patients have anxiety and depressive symptoms. A higher proportion of patients with multiple NCDs showed the evidence of symptoms of somatization, anxiety and depression compared to patients with single NCDs.

Prevalence of somatization symptoms was found to be 66.1% among the patients with NCDs which was higher than the available evidence^6^ where the prevalence of somatization was 35.1%. These differences in studies finding might be due to different criteria of sample selection and study settings. While observing the level of somatization among patients, 38.9% had mild, 21.4% had moderate and 5.8% had severe somatic symptoms.

The prevalence of anxiety symptoms was found to be 34.4% among our study participants in which 17.7%; 13.8 % and 2.9% of patients respectively had mild, moderate and severe anxiety disorders. This finding is slightly higher than the finding of the study conducted in India6in which 19.1% of patients had anxiety symptoms whereas our findings are almost similar with the study conducted in another study in India done by Verma et al. which showed 38.7% prevalence of anxiety among the cases.^14^ However, Taneja et al. showed a higher prevalence of depression (i.e. 56.7%) among NCDs cases.^4^ These studies finding might be varied due to the use of different data collection tool, characteristics of patients and data collection settings which might influence the prevalence of anxiety among patients.

In this study, the prevalence of depressive symptoms among NCDs patients was 41.0% which was found to be higher than the study done by Rijal et al. in 2016 in the general population of Nepal which showed 11.7% prevalence.^15^ It indicates that depression is higher among NCDs cases. However, available evidence showed varied results regarding the prevalence of depression among NCDs cases. The prevalence observed in our study is higher compared to other study done in India in which 29.1% of patients had depressive symptoms^6^ whereas lower than the study conducted in the urban slum of East Delhi which found depression among more than half (56.4%) of NCDs patients.^4^ The difference in findings might be due to difference in characteristics of samples included in the study. In our study included five types of NCDs patients whereas these two studies excluded patients with COPD and CKD in their studies.^4,6^ However, our observation in general are consistent with the prevalence of depression reported by Verma et al. which showed 58.1% of depression among NCDs patients.^14^ On the severity of depression, our study revealed 21.2% mild, 18.1 % moderate and 1.7% severe depression among the patients.

Our country is also giving priorities for non-communicable diseases and mental health through implementing sustainable developmental goal in national health policy. Nepal health research council, university grant commission and other NGOs have also mentioned NCDs and mental health as a priorities area for funding. Exploring psychiatric morbidities in patients with major non-communicable diseases using standard screening tool is the strong point of this study. Despite this, our study has certain limitations like- it was conducted in only one study setting and hospital admitted patients only, which may limit the generalizability of the study and it did not exclude the patients with other health problems which might have influenced the study findings.

## CONCLUSIONS

Psychiatric morbidities i.e. anxiety, depression and symptoms of somatization, is common among the patients suffering from non-communicable diseases such as diabetes, hypertension, chronic obstructive pulmonary disease, cardiovascular diseases and chronic kidney disease in Nepal. Anxiety and depression are proportionally higher in patients with DM and CKD than other NCDs patients. Likewise, patient with multiple NCDs tends to have higher psychiatric morbidities compared to patients with a single disease. Hence, there is a need to develop an integrated care model to manage these morbidities to the vulnerable groups at all level.
